# Economic Impact of Revision Operations for Adjacent Segment Disease of the Subaxial Cervical Spine

**DOI:** 10.5435/JAAOSGlobal-D-22-00058

**Published:** 2022-04-21

**Authors:** John Bonano, Daniel D. Cummins, Shane Burch, Sigurd H. Berven, Vedat Deviren, Christopher P. Ames, Bobby Tay, Aaron J. Clark, Alekos A. Theologis

**Affiliations:** From the Department of Orthopaedic Surgery (Dr. Bonano, Cummins, Dr. Burch, Dr. Berven, Dr. Deviren, Dr. Tay, and Dr. Theologis), and the Department of Neurological Surgery (Dr. Ames, and Dr. Clark), University of California–San Francisco (UCSF), San Francisco, CA.

## Abstract

Adjacent segment disease (ASD) of the cervical spine is a common disabling phenomenon that often requires surgical intervention. The goal of this study was to evaluate the economic impact of revision operations for cervical ASD.

**Methods:**

Consecutive adults who underwent revision cervical spine surgery for ASD at a single institution between 2014 and 2017 were retrospectively reviewed. Direct costs were identified from medical billing data and calculated for each revision surgery for ASD. Incomplete cost data for revision operations were used as a criterion for exclusion. Cost data were stratified based on the approach of the index and revision operations.

**Results:**

Eighty-five patients (average age 57 ± 10 years) underwent revisions for cervical ASD, which summed to $2 million (average $23,702). Revisions consisted of 45 anterior operations (anterior cervical diskectomy and fusion, 34; corpectomy, 10; and cervical disk arthroplasty, 1), 32 posterior operations (posterior cervical fusion, 14; foraminotomy, 14; and laminoplasty, 4), and 8 circumferential operations. Circumferential revisions had notably higher average direct costs ($57,376) than single approaches (anterior, $20,084 and posterior, $20,371). Of posterior revisions, foraminotomies had the lowest average direct costs ($5,389), whereas posterior cervical fusion had the highest average direct costs ($35,950). Of anterior revisions, corpectomies ($30,265) had notably greater average direct costs than anterior cervical diskectomy and fusion ($17,514). Costs were not notably different for revision approaches based on the index operations' approach.

**Discussion:**

Revision operations for cervical ASD are highly heterogeneous and associated with an average direct cost of $27,702. Over 3 years, revisions for 85 patients with cervical ASD represented a notable economic expense (greater than $2.0 million).

**Data availability:**

Deidentified data may be provided by request to the corresponding author.

Adjacent segment disease (ASD) is a relatively common sequelae of surgical intervention on the cervical spine.^1,2^ Its radiographic and clinical presentations are highly heterogeneous. Radiographic features may include degenerative disk disease, spondylolisthesis, and segmental kyphosis cranial and/or caudal to a prior fusion with or without instrumentation. Clinical symptoms may manifest as isolated neck pain, unilateral and/or bilateral radiculopathy, and/or myelopathy.^3^ Although nonsurgical measures hold great utility for managing cervical ASD, disabling symptoms often necessitate revision operations.

As revision operations for cervical ASD can be technically challenging and carry potentially greater risks than primary operations, much interest in the literature has focused on identifying risk factors for the development of cervical ASD^4,5,6,7^ and elucidating comparative utility of different surgical techniques on preventing the development of ASD (ie, anterior cervical diskectomy and fusion [ACDF] versus anterior cervical disk arthroplasty).^8,9,10,11,12,13^ The economic impact associated with revision operations for cervical ASD, however, remains relatively unexplored.

As the healthcare system in the United States transitions to a value-based economy, defining costs for episodes of care holds growing importance. Accurate cost estimates are also necessary to establish accurate payment thresholds for newer reimbursement models, including bundle payments.^14-16^ As such, the goal of this study was to evaluate the economic impact of revision operations for cervical ASD after previous cervical spine surgery.

## Methods

### Patient Cohorts

After approval was obtained by the institutional review board at our institution, consecutive adults (age >18 years) who underwent revision cervical spine surgery for subaxial cervical ASD (defined as radiographic changes at supra-adjacent and/or subjacent levels to a previous cervical spine operation resulting in new radiculopathy and/or myelopathy) at a single institution between 2014 and 2017 were retrospectively reviewed. Informed consent was not required, as it was deemed exempt from requirement by our institution's IRB. Data collected included patient demographics (age, sex, body mass index, and American Society of Anesthesiologists' score), surgical estimated blood loss, hospital length of stay, and cost data. Direct costs (surgical supplies/implants, room/care, pharmacy, and services) were identified from medical billing data and calculated for each revision surgery for ASD. Not included in direct cost data were charges, surgeon fees, or revision operations for indications other than ASD (ie, pseudarthrosis). No cost data were obtained for the index operations. Incomplete cost data for revision operations were used as a criterion for exclusion. Patients with index operations for tumors and infection were excluded. In addition, patients with index operations that included instrumentation distal to T2 and/or to the occiput were excluded.

The aforementioned data were analyzed for the entire cohort and were divided and compared between three subgroups based on the surgical approach used for the revision operation (group 1: anterior versus group 2: posterior versus group 3: AP [circumferential]). Cost data were also stratified based on the approach of the index and revision operations.

### Statistical Analysis

All statistical analyses were performed using Microsoft Excel. Chi-squared tests were used to compare noncontinuous variables between groups. Analysis of variance analyses were used to compare continuous variables between groups. A *P* value of <0.05 defined statistical significance.

## Results

**Figure 1:**
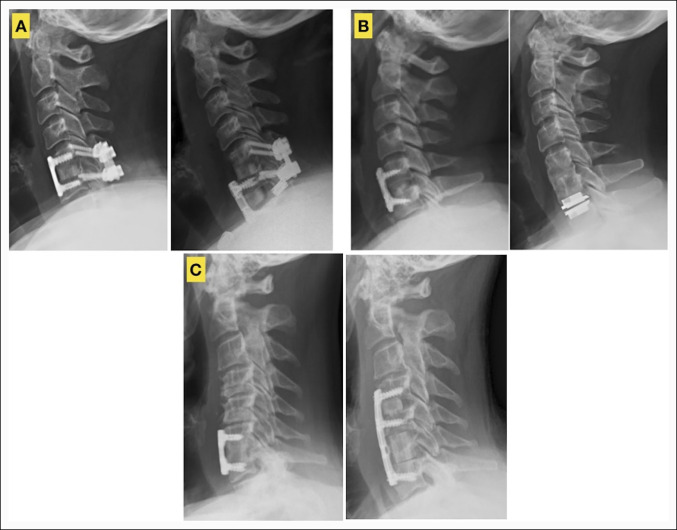
Representative preoperative and postoperative images of patients who underwent anterior operations to address cervical adjacent disease. **A**, Example of a patient who underwent removal of previous anterior plate from C5-6 and distal adjacent segment ACDF with revision C6-7 anterior plating. **B**, Example of a patient who underwent removal of previous anterior plate from C5-6 and distal adjacent segment cervical disk arthroplasty at C6-7. **C**, Example of a patient who underwent removal of previous anterior plate from C6-7 and revision C4-5 ACDF, C6 corpectomy, and revision C4-7 anterior plating. ACDF = anterior cervical diskectomy and fusion

**Figure 2:**
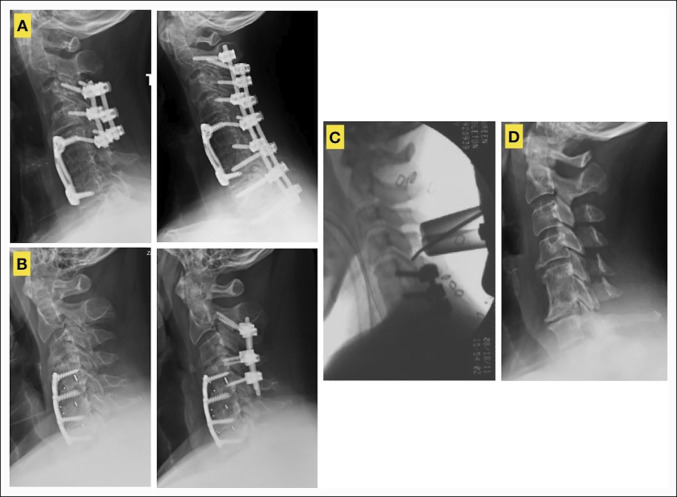
Representative images of patients who underwent posterior operations to address cervical adjacent disease. **A**, Example of a patient who underwent removal of previous posterior instrumentation from C3 to C5 and extension of posterior instrumentation from C2 to T2. **B**, Example of a patient who underwent C2 to C5 posterior instrumentation for adjacent segment disease above a prior C4-7 ACDF. **C**, Example of a patient who underwent unilateral C4-5 laminoforaminotomy for symptomatic adjacent segment disease cranial to a prior C5-7 circumferential operation. **D**, Example of a patient who underwent a C3-6 laminoplasty for cranial and caudal adjacent segment disease after a prior C5-6 ACDF. ACDF = anterior cervical diskectomy and fusion

**Figure 3:**
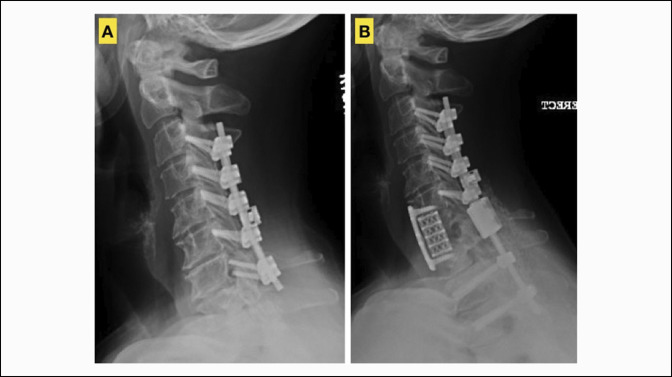
Example of preoperative (**A**) and postoperative (**B**) images of a patient who underwent a circumferential operation for cervical adjacent segment disease. Specifically, this patient underwent a C7 anterior corpectomy with C6-T1 anterior plating and extension of prior C3-6 posterior instrumentation to T3 for distal adjacent segment disease.

Eighty-five patients (average age 57 ± 10 years) met inclusion criteria and were included for analysis. Patient demographics are presented in Table 1. The group consisted primarily of patients who underwent anterior revision operations (n = 45) (Figure 1) and posterior revision operations (n = 32) (Figure 2). Few patients (n = 8) underwent revision operations that involved a circumferential approach (Figure 3). Revisions consisted of 45 anterior operations, 32 posterior operations, and 8 circumferential operations. Of the 45 anterior operations, the most commonly performed operation involved an ACDF (n = 34) (Figure 1). ACDFs were performed at 1 level (n = 13), 2 levels (n = 10), 3 levels (n = 9), and 4 levels (n = 2). Anterior corpectomies (n = 10) and cervical disk arthroplasties (n = 1) were the minority of anterior revisions (Figure 1). Number of corpectomy levels consisted of 1 level (n = 5), 2 levels (n = 4), and 3 levels (n = 1). Of the 32 posterior operations, posterior cervical fusions (PCF)/laminectomies (lami) (n = 14) and laminoforaminotomies (n = 14) were most common (Figure 2). There were a variety of posterior cervical fusion levels performed: 1 level (n = 2), 2 levels (n = 1), 3 levels (n = 2), 4 levels (n = 1), 5 levels (n = 0), 6 levels (n = 3), 7 levels (n = 3), 8 levels (n = 1), and 9 levels (n = 1). The least common revision posterior operation was laminoplasty (n = 4) (Figure 2). All laminoplasty operations were 3 levels.

**Table 1:** Comparison of Three Patient Cohorts by the Surgical Approach for the Treatment of Cervical Adjacent Segment Disease

	All	Anterior	Posterior	Circumferential	*P*
N	85	45	32	8	n/a
Age (avg ± SD; range)	57 ± 10 (23-80)	57 ± 9 (40-74)	56 ± 12 (23-80)	64 ± 8 (48-70)	0.19
Gender					0.15
Male	36	14	17	3	
Female	49	31	15	5	
BMI (avg ± SD; range)	29.6 ± 5.8 (18.6-45)	28.9 ± 5.8 (18.6-45)	30.2 ± 5.6 (20-40)	31 ± 6.6 (24.5-42)	0.55
ASA score (avg ± SD; range)	2.2 ± 0.56 (1-4)	2.1 ± 0.47 (1-3)	2.3 ± 0.58 (1-3)	2.7 ± 0.76 (2-4)	0.04
EBL (mL) (avg ± SD; range)	257 ± 383 (20-2,400)	146 ± 159 (20-750)	378 ± 541 (20-2,400)	550 ± 431 (100-1,250)	<0.01
LOS (days) (avg ± SD; range)	3.7 ± 4.2 (0-29)	3.3 ± 2.7 (1-12)	2.7 ± 2.8 (0-10)	10.3 ± 8.1 (4.8-7.0)	<0.01

ASA = American Society of Anesthesiologists, BMI = body mass index, EBL = estimated blood loss, LOS = hospital length of stay

For revision operations based on the approach of the index operation, there were 33 patients who underwent revision anterior operations after an initial anterior operation (ACDF, 24; corpectomy, 8; and cervical disk arthroplasty, 1). There were 21 patients who underwent revision posterior operations after an initial anterior operation (PCF/lami, 7; bilateral laminoforaminotomy, 3; unilateral laminoforaminotomy, 7; and laminoplasty, 4). There were five patients who underwent circumferential revisions after an initial anterior operation. After an index posterior operation, revisions for ASD included anterior (n = 11; ACDF, 9 and corpectomy, 2), posterior (n = 6; PCF/lami, 4; bilateral laminoforaminotomy, 4; and unilateral laminoforaminotomy, 4), and circumferential (n = 2). After an index circumferential operation, revisions for ASD included anterior (n = 1; ACDF, 1), posterior (n = 5; PCF/lami, 3 and unilateral laminoforaminotomy, 2), and circumferential (n = 1).

Comparative analyses found that there were no statistical differences in average age, sex representation, and body mass index between patients who underwent anterior-only, posterior-only, and circumferential operations (Table 1). Patients whose revision operations involved a circumferential approach had notably higher preoperative American Society of Anesthesiologists' scores, notably greater intraoperative estimated blood loss, and notably longer hospital lengths of stay compared with revision operations performed through anterior-only approaches and posterior-only approaches (Table 1).

The direct cost for the entire cohort of 85 patients was $2 million. The average direct cost per case was $23,702. Revisions that entailed a circumferential approach had notably higher average direct costs ($57,376 ± 31,258) than revisions performed through an anterior-only approach ($20,084 ± 9,879) and posterior-only approach ($20,371 ± 16,898) (Table 2). While costs varied if the circumferential revision was performed after an index anterior operation ($53,889), index posterior operation ($82,952), or index circumferential operation ($23,655), statistical comparisons could not be performed because the number of patients was too small (Table 3).

**Table 2:** Average Direct Costs for Revision Operations by the Approach for Cervical Adjacent Segment Disease

Anterior Revisions (n = 45)	All	ACDF (n = 34)	Corpectomy + ASF (n = 10)	CDA (n = 1)	*P*	
Direct costs ($)	20,084 ± 9,879	17,514 ± 4,838	30,265 ± 17,071	17,319	<0.01	

Posterior Revisions (n = 32)	All	PCF/lami (n = 14)	B/L LFo (n = 4)	Unilateral LFo (n = 10)	Laminoplasty (n = 4)	*P*
Direct costs ($)	20,371 ± 16,898	35,950 ± 13,477	5,389 ± 3,009	6,563 ± 4,146	15,344 ± 1,282	<0.01

Circumferential Revisions (n = 8)	All					
Direct costs ($)	57,376 ± $31,258	—	—	—	—	—

ACDF = anterior cervical diskectomy and fusion, ASF = anterior spinal fusion, B/L = bilateral, CDA = cervical disk arthroplasty, LFo = laminoforaminotomy, PCF = posterior cervical fusion

**Table 3:** Average Direct Costs for Revision Operations for Cervical Adjacent Segment Disease by the Index Operations' Approach

Factor	Index Approach			*P*
	Anterior	Posterior	360	
Anterior revisions				
All	20,378 ± 10,8016 (n = 33)	19,971 ± 6,895 (n = 11)	11,575 (n = 1)	n/a
ACDF	17,016 ± 3,696 (n = 24)	19,524 ± 7,105 (n = 9)	11,575 (n = 1)	n/a
Corpectomy + ASF	30,850 ± 18,012 (n = 8)	21,985 ± 7,856 (n = 2)	—	n/a
CDA	17,319 (n = 1)	n/a (n = 0)	—	n/a
*p*	<0.01	n/a	n/a	
Posterior revisions				
All	17,376 ± 15,308 (n = 21)	25,325 ± 20,835 (n = 6)	27,005 ± 18,960 (n = 5)	0.71
PCF/lami	35,321 ± 12,812 (n = 7)	35,879 ± 16,672 (n = 4)	37,514 ± 16,313 (n = 3)	0.83
B/L LFo	5,863 ± 3,497 (n = 3)	3,965 (n = 1)	n/a (n = 1)	n/a
Unilateral LFo	5,526 ± 1,903 (n = 7)	4,470 (n = 1)	37,514 ± 16,313 (n = 2)	n/a
Laminoplasty	15,344 ± 1,282 (n = 4)	n/a (n = 0)	n/a (n = 0)	n/a
*p*	<0.01	n/a	n/a	
Circumferential revisions				
All	53,889 ± 25,548 (n = 5)	82,952 ± 41,504 (n = 2)	23,655 (n = 1)	n/a

ACDF = anterior cervical diskectomy and fusion, ASF = anterior spinal fusion, B/L = bilateral, CDA = cervical disk arthroplasty, LFo = laminoforaminotomy, PCF = posterior cervical fusion

Of the anterior revisions, ACDFs had notably lower average direct costs compared with anterior corpectomies ($17,514 versus $30,265) (Table 2). The one cervical disk arthroplasty case had similar direct costs to ACDFs ($17,319 versus $17,514) (Table 2). Average costs were similar if the anterior revision was performed after an index anterior operation ($20,378), index posterior operation ($19,971), or index circumferential operation ($11,575) (Table 3).

Of the posterior revisions, unilateral laminoforaminotomies ($6,563) and bilateral laminoforaminotomies ($5,389) had the lowest average direct costs, whereas PCF/laminectomies were associated with the greatest average direct costs ($35,950) (Table 2). The four laminoplasty cases had an average direct cost of $15,344 (Table 2). Average costs were statistically similar if the posterior revision was performed after an index anterior operation ($17,376), index posterior operation ($25,325), or index circumferential operation ($27,005) (Table 3).

## Discussion

Revision operations for ASD in the cervical spine are highly heterogeneous. In the manuscript presented heretofore, we present direct cost data for 85 revision operations to address ASD of the cervical spine at a single institution. This study has five major findings: (1) direct costs for all operations summed to $2 million with an average of $23,702 per case; (2) circumferential operations had notably higher costs than single approaches; (3) of anterior revisions, corpectomies had notably greater costs than ACDFs; (4) of posterior revisions, foraminotomies had the lowest costs, whereas PCF/lami had the greatest costs; and (5) costs of revision operations were not notably different based on the index operations' approach. To the authors' knowledge, this is the first study to report the cost of revision cervical surgery for ASD. It is also the first study to report the cost differential between surgical approaches to treat cervical ASD. These results complement and add a new dimension to the growing literature on ASD in the cervical spine.

Previous studies evaluating costs of operations in the cervical spine have focused on costs of primary operations and their relative cost effectiveness.^17,18,19,20,21,22,23,24,25,26,27,28,29,30^ In these studies, direct costs for the individual operations ranged: ACDF ($8,192 to $20,014), cervical disk arthroplasty ($9,999 to $11,472), posterior cervical laminoforaminotomy ($3,570 to $4,320), laminoplasty ($15,426), and posterior cervical fusion ($17,740 to $32,125).^18,19^ Comparing these data with our cost data reveals several interesting similarities and differences. Similar to our data, the aforementioned studies demonstrated that posterior laminoforaminotomies had the lowest direct costs, ACDFs and cervical disk arthroplasty had similar direct costs, and posterior cervical fusions had the highest direct costs. Although magnitude of the direct costs for laminoplasty is similar to our results, our average costs and maximum costs for revision operations of ACDFs, cervical disk arthroplasty, laminoforaminotomies, and posterior cervical fusions are notably higher than the previously reported costs for primary operations. These differences may reflect variability in a variety of perioperative factors, including institutional implant costs, operating room costs, surgical time, and postoperative care protocols. Alternatively, our higher cost data may be a consequence of the added complexity associated with revision operations to address ASD relative to primary operations. Our data on length of stay, particularly for anterior revisions (average 3.3 days, max 12 days) and circumferential operations, highlight the added complexity of these ASD revision operations relative to primary operations for degenerative cervical pathology. That revision operations for cervical ASD are associated with higher direct costs suggest that they should be considered differently from a reimbursement perspective. In addition, because revisions are more expensive than the index operation, continued research into the causes of ASD and surgical techniques to prevent ASD are critical for limiting overall costs of care.

Our results should be considered within the context of its limitations. As a retrospective analysis and a single institution's experience, our results may not be representative of the larger spine community. For example, costs associated with surgery in this study are only reflective of our institution and likely not others because costs of surgeries (including implants) vary tremendously between different surgeons and hospital systems (rural versus metropolitan centers). Furthermore, the cost of the same operation can substantially vary by surgeon, based on the expense, type, and quantity of implants used. However, the considerable heterogeneity of the operations in this study mirrors the variety seen in clinical practice and likely makes the results relatively generalizable. In addition to the heterogeneity in our cohort, sampling of a 3-year period introduces the chance that independent perioperative and intraoperative factors, including variations in operating room protocols, surgical instrumentation, differences in the trainee skill level, and postoperative pain regimens and/or rehabilitation protocols, may have influenced our cost data. Another limitation is that our comparison of direct costs relative to those of primary operations is reliant on previous literature because we were not able to provide direct cost data for our patients' index operations (ie, the operation was performed at another hospital or before our institution's collection of cost data). In addition, we did not assess health-related quality of life outcome scores or measure radiographic parameters. However, these are not felt to contribute to cost. As health-related quality of life outcome scores were not consistently available, our data do not offer commentary on the relative utility and effectiveness of one surgical technique over another for addressing cervical ASD. Instead, we advocate for a surgical approach that addresses the patient's symptoms and radiographic pathology based on the individual surgeon's preference. Although our data are neither equipped nor intended to serve as a cost-effectiveness analysis, they do importantly set the foundation for future studies focused on determining the cost effectiveness of revision operations for cervical ASD.

In summary, revision operations for ASD in the cervical spine are highly heterogeneous and were associated with an average direct cost of $27,702. Over a 3-year period at a single tertiary referral center, surgical care for 85 patients with cervical ASD represented a notable economic expense (greater than $2.0 million). As cost is an important metric for each episode in the continuum of care of patients with cervical ASD and for defining accurate payment thresholds and reimbursements in our healthcare system, our results will ideally motivate and facilitate new health economic analyses on revision operations for cervical ASD.
